# From Strong Fluoride Binding to Reversible Electrodesorption: S, N-Regulated La-MOF-Derived Carbon Electrodes for Capacitive Deionization Defluoridation

**DOI:** 10.3390/ma19122556

**Published:** 2026-06-12

**Authors:** Xue Yang, Shirong Yang, Dongbao Song, Hongtao Zhang, Junfeng Li, Pu Wang

**Affiliations:** 1 College of Water Conservancy and Architectural Engineering, Shihezi University, Shihezi 832000, China; yangxue@stu.shzu.edu.cn (X.Y.); yangshirong@stu.shzu.edu.cn (S.Y.); songdongbao94@163.com (D.S.); hongtao.zhang@shzu.edu.cn (H.Z.); 2 Water Resources and Water Environment Engineering Technology Center, Xinjiang Key Laboratory of Synthesis and Application of Carbon Nanomaterials, School of Civil Engineering, Kashi University, Kashi 844000, China

**Keywords:** capacitive deionization, fluoride removal, derived carbon, electrical driving forces, La_2_O_2_S/g-C_3_N_4_

## Abstract

**Highlights:**

- S, N co-regulated La_2_O_2S_/g-C_3_N_4_-derived carbon electrodes were successfully fabricated via thiourea-assisted carbonization of La-BDC-140.
- La-CNS_3_ achieved a high fluoride removal capacity of 195 mg·g^−1^ at an initial fluoride concentration of 100 mg·L^−1^.
- Reverse-voltage regeneration enabled reversible fluoride (F^−^) electrosorption without chemicals.

**What are the main findings?**

- Under electric-field stimulation, the electrode exhibited excellent reversible adsorption/desorption, with a regeneration efficiency of F^−^ exceeding 70%.
- Mechanistic analyses revealed that fluoride removal is mainly governed by La-F coordination, surface hydroxyl/water ligand exchange, and interfacial charge redistribution.
- DFT calculations further confirmed that the La_2_O_2_S/g-C_3_N_4_ structure provides a favorable balance between fluoride adsorption strength and desorption reversibility.

**What are the implications of the main findings?**

- These findings demonstrate that S, N co-regulation is an effective strategy to address the regeneration limitations of conventional La-based defluoridation materials.
- The La_2_O_2_S/g-C_3_N_4_-derived carbon electrode enables high-capacity, selective, and electrically regenerable fluoride removal in capacitive deionization (CDI) systems.
- This work provides a theoretical basis for designing sustainable rare-earth-based electrodes with balanced fluoride adsorption strength and desorption reversibility.

**Abstract:**

La-MOFs exhibit strong affinity toward anions such as F^−^ and phosphate. However, conventional La-MOFs show limited regeneration performance when used as CDI electrodes, posing a major challenge for practical applications. In this study, a high-performance sulfur and nitrogen co-doped La-BDC-140-derived carbon electrode (La-CNS_3_) was fabricated via a coupled carbonization and doping strategy. The optimized La-CNS_3_ electrode possessed abundant defects, a mesoporous structure, favorable hydrophilicity, and rapid charge-transfer capability, which collectively enhanced fluoride electrosorption. At 1.4 V, La-CNS_3_ achieved a fluoride removal capacity of 31.86 mg·g^−1^ for 10 mg·L^−1^ F^−^ solution and up to 195 mg·g^−1^ at an initial F^−^ concentration of 100 mg·L^−1^. More importantly, partial fluoride desorption was realized solely under reverse voltage, and the electrode maintained favorable defluoridation performance over 50 adsorption–desorption cycles. In actual groundwater treatment, the effluent fluoride concentration decreased to below 1.0 mg·L^−1^ after 120 min. XPS analysis and DFT calculations revealed that fluoride removal was mainly governed by La-F coordination, surface hydroxyl/water ligand exchange, and interfacial charge redistribution. The La_2_O_2_S/g-C_3_N_4_ structure provided a favorable balance between fluoride adsorption strength and desorption reversibility. This work offers a promising strategy for designing efficient, selective, and electrically regenerable rare-earth-based CDI electrodes for fluoride-contaminated water treatment.

## 1. Introduction

Fluoride is a naturally occurring inorganic ion widely present in natural waters. At an appropriate concentration, fluoride in drinking water can help prevent dental caries. However, excessive fluoride levels, particularly those originating from fluoride-rich groundwater or industrial wastewater, may pose serious risks to human health. Long-term intake of high-fluoride water can lead to dental fluorosis and skeletal fluorosis, depending on the exposure dose and duration [1,2]. The World Health Organization (WHO) recommends a guideline value of 1.5 mg·L^−1^ for fluoride in drinking water. Nevertheless, in many regions affected by geogenic fluoride enrichment, fluoride concentrations in groundwater exceed this guideline value, thereby threatening the safety of local drinking-water supplies [3]. Therefore, developing efficient, sustainable, and cost-effective defluoridation technologies is of great significance for protecting public health and ensuring water resource security.

Currently, the major technologies for fluoride removal from water include chemical precipitation, ion exchange, membrane separation, and adsorption. Chemical precipitation removes F^−^ by adding lime or aluminum salts to form fluoride-containing precipitates. Although this method is simple to operate and relatively inexpensive, it generates large amounts of sludge and shows limited efficiency for low-concentration F^−^ removal [4]. Ion exchange relies on strongly acidic or strongly basic resins to capture F^−^ from water and exhibits relatively high selectivity toward fluoride. Nevertheless, its performance is easily affected by coexisting anions, and the resins often require frequent regeneration with substantial consumption of chemical reagents [5]. Membrane-based technologies, such as nanofiltration and reverse osmosis, can effectively remove F^−^, but their practical application in large-scale water treatment is restricted by high capital costs, considerable energy consumption, and severe membrane fouling [6]. In contrast, adsorption has attracted extensive attention because of its operational simplicity, low energy demand, and potential recyclability. However, conventional adsorbents, such as activated carbon and alumina, usually exhibit limited adsorption capacity and insufficient selectivity when treating waters containing low levels of F^−^ [7].

CDI, an emerging electrically driven water treatment technology, removes dissolved ions through electrostatic adsorption at the electrode–solution interface. CDI offers several advantages, including low energy consumption, mild operating conditions, and facile regeneration, making it particularly suitable for efficient F^−^ removal from low-salinity water systems [8,9]. However, traditional carbon-based electrodes generally show weak selective adsorption toward F^−^, and their adsorption capacity, adsorption rate, and cycling stability still require substantial improvement [10]. Therefore, the development of electrode materials with high F^−^ selectivity and reversible adsorption capability is critical for achieving efficient fluoride removal by CDI.

Rare-earth metals have been widely applied in water treatment owing to their unique electronic structures and strong coordination abilities. In particular, La^3+^ can form stable coordination complexes with F^−^, enabling efficient fluoride capture [11,12]. In recent years, La-based metal–organic frameworks, namely La-MOFs, have received increasing attention in defluoridation studies because of their high specific surface area, tunable pore structure, and abundant surface active sites. Previous studies have demonstrated that conventional La-MOFs can exhibit high initial adsorption capacities. However, research on the regeneration of La-MOF-based adsorbents remains limited. Existing reports indicate that pristine La-MOF adsorbents often suffer from unsatisfactory cycling performance. For example, the La-MOF prepared by Zhang et al. retained only 71% of its fluoride removal performance after three adsorption–desorption cycles [13]. Jiang et al. synthesized a porous LaMOF-20%-C material via pyrolysis for phosphate removal from water. To evaluate its reusability in practical applications, desorption was conducted using Na_2_CO_3_ solution at 50 °C. The adsorption capacity decreased slightly with increasing cycle number, suggesting that some active sites on the LaMOF-20%-C surface were irreversibly occupied, thereby making regeneration difficult and reducing its adsorption capacity. After four adsorption–desorption cycles, the adsorption capacity was maintained at 66% [14]. To regenerate La/Fe-MOFs-NH_2_, Wei et al. employed 0.5 mol·L^−1^ NaOH solution, but the adsorption efficiency decreased to below 90% after five cycles [15]. Although La-800 exhibits excellent defluoridation performance, the deintercalation of F^−^ still requires the synergistic effect of alkaline treatment and an electric field. Therefore, achieving the regeneration of La-MOF-derived electrode materials solely under electric-field driving remains a key challenge.

Recent studies have shown that heteroatom doping is an effective strategy for regulating the electronic structure and enhancing the adsorption capability of MOF-derived carbon materials, and it has therefore attracted broad interest in water treatment applications [16]. The co-doping of nonmetallic heteroatoms, such as B, N, S, P, and halogens, into the sp^2^-hybridized graphitic lattice of MOF-derived carbon materials can optimize the surface electronic distribution and polarity by exploiting the electronegativity differences between carbon and heteroatoms, as well as their chemical configurations within the carbon matrix. This process can increase the number of active sites and improve interfacial adsorption behavior [17]. Moreover, heteroatom doping can enhance the response of carbon-based materials to an external electric field, enabling partial desorption of adsorbed F^−^ under reverse voltage and thereby improving electrochemical regeneration efficiency [18,19].

Based on the above considerations, this study focuses on the following aspects. First, a high-performance S, N co-doped La-BDC-140-derived carbon electrode (La-CNS_3_), was prepared through a coupled carbonization and doping strategy. Its physicochemical properties and electrochemical performance were systematically characterized, and its highly selective adsorption toward F^−^ in CDI was verified. Second, electrochemical measurements and fluoride removal experiments were conducted to elucidate the strong affinity of the derived carbon electrode for F^−^ ions and its selective adsorption mechanism. Third, by combining characterizations of the La-CNS_3_ electrode before and after defluoridation with density functional theory calculations, including adsorption energy, charge density distribution, and F^−^ migration energy barriers, the regeneration mechanism of the electrode under reverse voltage was investigated. This work clarifies the mechanism of electric-field-driven reversible F^−^ adsorption, provides a theoretical basis for voltage-driven regeneration of MOF-derived carbon electrodes, and offers guidance for the design and application of regenerable rare-earth-based defluoridation materials.

## 2. Materials and Methods

### 2.1. Material Synthesis

Lanthanum nitrate hexahydrate (La(NO_3_)_3_·6H_2_O, 99%), terephthalic acid (H_2_BDC-NH_2_, 98%),N, N-dimethylformamide (DMF, 99.5%), thiourea (CH_4_N_2_S, 99%), ethyl alcohol (EtOH, 99.5%), carbon black, polyvinylidene fluoride (PVDF, Mw~400,000) and N- methylpyrrolidone (NMP, 99.5%) were all purchased from Aladdin Biochemical Technology Co., Ltd. ((Shanghai, China). All chemical reagents were not further purified. In addition, all solutions were prepared with purified water produced by Wahaha Group Co., Ltd. (Hangzhou, China) (Resistivity: 18.25 Ω cm^−1^).

**Figure 1:**
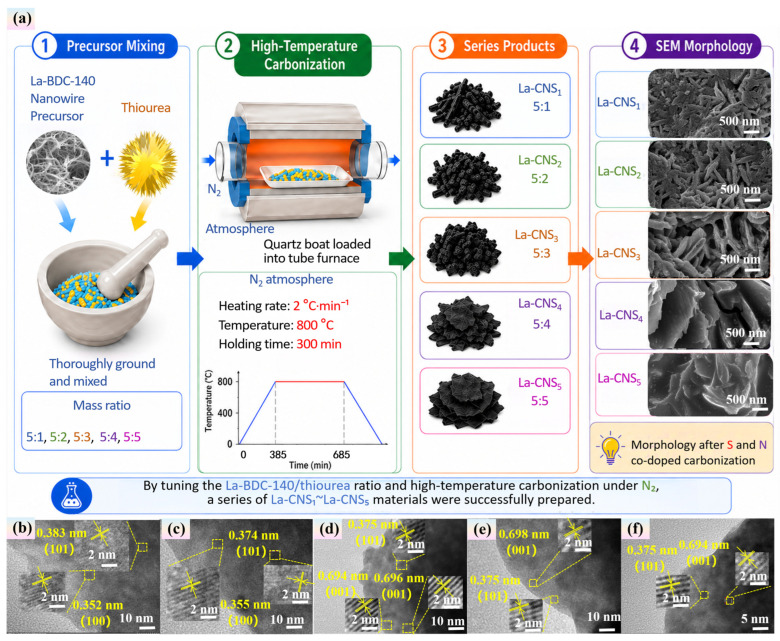
(**a**) Synthesis process of La-CNS_x_ and SEM images, high-resolution TEM images of (**b**) La-CNS_1_, (**c**) La-CNS_2_, (**d**) La-CNS_3_, (**e**) La-CNS_4_ and (**f**) La-CNS_5_.

The synthesis procedure of La-BDC-140 was optimized and improved based on our previous studies [20]. A certain amount of La-BDC-140 was thoroughly ground and mixed with thiourea in a mortar at mass ratios of 5:1, 5:2, 5:3, 5:4, and 5:5. The resulting mixtures were then transferred into quartz boats and placed in a tubular furnace under an N_2_ atmosphere. Based on the results obtained in the previous chapter, the temperature was increased to 800 °C at a heating rate of 2 °C·min^−1^ and maintained for 300 min. The obtained materials were denoted as La-CNS_x_ and specifically labeled as La-CNS_1_, La-CNS_2_, La-CNS_3_, La-CNS_4_, and La-CNS_5_, respectively (Figure 1).

### 2.2. Electrode Preparation

The electrode slurry was fabricated by dispersing the corresponding active materials in N-methyl-2-pyrrolidone (NMP). La-CNS_x_ and PVDF were mixed at a mass ratio of 9:1, followed by continuous stirring in NMP until a uniform slurry was obtained. The prepared slurry was subsequently cast onto carbon paper using a film applicator with a coating thickness of 0.1 mm. The resulting electrodes had dimensions of 3.5 cm × 3.5 cm, with an active layer thickness of approximately 75 μm. After coating, the electrodes were vacuum-dried at 60 °C for 12 h to yield La-CNS_x_-based electrodes. As a control, activated carbon (AC) electrodes were prepared following the same protocol and with identical electrode dimensions, using AC as the active component. To balance the mass loading between the anode and cathode, the mass of each electrode was maintained at approximately 15~20 mg, as illustrated in Figure S1a.

### 2.3. Material Characterization

The crystalline structure, morphology, and physicochemical characteristics of the as-prepared samples were comprehensively analyzed using multiple characterization methods. The surface morphology and spatial elemental distribution were characterized by scanning electron microscopy coupled with energy-dispersive X-ray spectroscopy (SEM-EDS, Zeiss Gemini 360; Ultim Max 40, Oberkochen, Germany). Transmission electron microscopy (TEM, JEOL JEM-120i, Tokyo, Japan) was further employed to reveal the fine microstructural features. Powder X-ray diffraction (XRD, Bruker D2 Phaser, Karlsruhe, Germany) was conducted within a 2θ range of 5°~90° to determine the phase composition and structural changes of the materials. Fourier-transform infrared spectroscopy (FTIR, Thermo Fisher Scientific Nicolet iS20, Waltham, MA, USA) was recorded in attenuated total reflectance (ATR) mode over the range of 400~4000 cm^−1^ to identify surface functional groups. Raman spectroscopy (Horiba LabRAM HR Evolution, Kyoto, Japan) was employed to evaluate the graphitization degree, defect density, and structural disorder of the carbon materials.

The pore structure and textural parameters were evaluated by N_2_ adsorption–desorption analysis using a BELSORP MAX X system (MicrotracBEL Corp., Osaka, Japan). The specific surface area was calculated based on the Brunauer–Emmett–Teller (BET) equation, whereas the pore-size distribution was obtained using the density functional theory (DFT) model with BELControl software, version 1.4.4. X-ray photoelectron spectroscopy (XPS, Thermo Scientific K-Alpha, Waltham, MA, USA) was used to investigate the surface elemental composition and corresponding chemical states. Raman spectroscopy was performed on a spectrometer (LabRAM HR Evolution, HORIBA Scientific, Palaiseau, France) with a 532 nm laser source to analyze the carbon framework and molecular structural features of the samples. The surface wettability was assessed through static contact-angle measurements using a SZ-CAMC32 goniometer (Shanghai Xuanzhun (Shanghai, China)) based on the sessile-drop method, with each sample tested three times to ensure reproducibility. Thermogravimetric analysis (TGA, Netzsch STA 449 F3, Selb, Germany) was performed from 30 to 1000 °C at a heating rate of 5 °C·min^−1^ to evaluate the thermal stability and mass-loss characteristics of the samples. The concentration of F^−^ was measured using a fluoride ion analyzer equipped with a fluoride ion-selective electrode (pH/ION 3310 and F-800, Wissenschaftlich-Technische Werkstätten, WTW, Weilheim, Germany).

### 2.4. Electrochemical Testing

Electrochemical tests were conducted using electrodes prepared following the reported protocol for La-BDC-based electrodes [20]. All electrochemical measurements were carried out in a standard three-electrode configuration, where the prepared active-material electrode was used as the working electrode, platinum foil served as the counter electrode, and an Ag/AgCl electrode was employed as the reference electrode. A 1.0 mol·L^−1^ NaF aqueous solution was used as the electrolyte, and all measurements were performed on a CHI760E electrochemical workstation. Cyclic voltammetry (CV) was performed within a potential window of −1.0 to 1.0 V at scan rates of 1, 5, 10, 20, 50, and 100 mV·s^−1^ to record the current–potential response and evaluate the capacitive behavior of the electrodes. Galvanostatic charge–discharge (GCD) tests were conducted over the same potential range at current densities of 0.2, 0.4, 0.6, 0.8, 1.0, and 1.2 A·g^−1^ to assess the charge-storage performance and cycling stability of the electrode materials. Electrochemical impedance spectroscopy (EIS) was carried out over a frequency range of 100 kHz to 0.01 Hz under a small-amplitude sinusoidal AC signal, and the resulting impedance spectra were fitted using ZView 3 software. In addition, the charge-storage kinetics were analyzed by examining the relationship between the CV peak current and scan rate. The dependence of peak current on scan rate provides insight into the dominant electrochemical storage mechanism. A b-value close to 0.5 indicates a diffusion-controlled process, typically corresponding to battery-type behavior, whereas a b-value approaching 1.0 suggests a surface-controlled capacitive process. When the b-value falls between 0.5 and 1.0, both diffusion-controlled and capacitive processes contribute to charge storage. The detailed calculation method is described in the relevant references [21,22,23,24].

(1) $$i_{\mathrm{Total}}=av^{b}\;=\;i_{\mathrm{Capacitive}}\;+\;i_{\mathrm{Diffusion}}\;=\;k_{1}v\;+\;k_{2}v^{\frac{1}{2}}$$

In these equations, $i_{\mathrm{Total}}$ denotes the peak current measured at different scan rates, with the unit of mA. The parameters a and b are empirical constants obtained from fitting, while v represents the scan rate in mV·s^−1^. $i_{\mathrm{Capacitive}}$ is the current contribution arising from capacitive, surface-controlled processes, whereas $i_{\mathrm{Diffusion}}$ represents the current contribution associated with diffusion-controlled behavior; both are expressed in mA. The coefficients $k_{1}$ and $k_{2}$ are fitting parameters determined at a specific potential.

### 2.5. CDI Defluoridation Experiments

The CDI defluorination system is illustrated in Figure S1b. All fluoride removal tests were performed under a continuous recirculating mode. In a typical experiment, 50 mL of fluoride-containing solution with an initial F^−^ concentration of 10 mg·L^−1^, except for tests designed to investigate the effect of initial concentration, was introduced into the feed tank and circulated through the CDI unit using a peristaltic pump. The F^−^ concentration was monitored using a WTW fluoride ion analyzer. During the adsorption process, a constant voltage was supplied by an external power source, and the corresponding current/voltage responses were recorded in real time.

### 2.6. Calculations and Data Analysis

The specific capacitance (C, F·g^−1^) of the derived carbon electrode was calculated from the CV curves according to Equation (2).

(2) $$C=\frac{\int_{V_{a}}^{V_{c}}Idv}{2mv(V_{c}-V_{a})}$$

where *I* (A) is the instant current, *v* (V·s^−1^) is the potential scan rate, *m* (g) is the mass of the electrode, and *V*_c_ (V) and *V*_a_ (V) represent the upper and lower potential limits during the CV measurement, respectively.

The F^−^ removal capacity (FRC, mg·g^−1^) was calculated using Equation (3):

(3) $$\mathrm{FRC}\;=\;\frac{\left(C_{0}-C\right)V}{m}$$

where *C*_0_ and *C* (mg·L^−1^) represent the F^−^ concentrations in water after electrosorption, respectively, *V* (L) and *m* (mg) are respectively the volumes of the treated water and the total mass of electrode materials on both the anode and cathode.

The adsorption kinetic behavior was further evaluated using the nonlinear forms of the pseudo-first-order model (Equation (4)) and pseudo-second-order model (Equation (5)).

(4) $$q_{t}=q_{e}(1-e^{-k_{1}t})$$

(5) $$q_{t}=\frac{q_{e}^{2}k_{2}t}{1+q_{e}k_{2}t}$$

where *q*_t_ and *q*_e_ (mg·g^−1^) denote the adsorption capacities at time *t* (min) and at equilibrium, respectively. *k*_1_ (min^−1^) and *k*_2_ (mg·g^−1^·min^−1^) represent the apparent rate constants of the pseudo-first-order and pseudo-second-order models, respectively.

The adsorption isotherm data were fitted using the nonlinear forms of the Langmuir model (Equation (6)) and the Freundlich model (Equation (7)).

(6) $$q_{e}=\frac{q_{m}K_{L}C_{e}}{1+K_{L}C_{e}}$$

(7) $$q_{e}=K_{F}C_{e}^{\frac{1}{n}}$$

where *q*_e_ represents the equilibrium adsorption capacity (mg·g^−1^), *q*_m_ is the maximum adsorption capacity (mg·g^−1^), *K*_L_ is the Langmuir constant associated with the adsorption free energy (L·mg^−1^), *K*_F_ is the Freundlich constant related to the adsorption capacity, while n is an empirical parameter reflecting the heterogeneity of adsorption sites.

Charge efficiency (CE, %) is an important parameter reflecting the degree of charge utilization in a CDI system. Specific energy consumption (SEC, kWh·kg^−1^) is one of the key indicators for evaluating the energy-utilization efficiency of the system, and is mainly used to quantify the electrical energy consumed per unit amount of fluoride removed. The two parameters were calculated using the following equations [25,26].

(8) $$CE=\frac{60\times F\times n_{s}}{\int_{0}^{t}i\mathrm{dt}}$$

(9) $$SEC=\frac{U\times \int_{0}^{t}i\mathrm{dt}}{(C_{0}-C_{t})\times V}$$

In these equations, *F* represents the Faraday constant (96,485 C·mol^−1^); n_s_ is the amount of adsorbed salt (mol); *i* is the current during the charging process (A); $\int_{0}^{t}i\mathrm{dt}$ is the total input charge (C); and *V* represents the voltage window during fluoride removal (V).

## 3. Results

### 3.1. The Physical and Chemical Properties of the Materials

The thiourea doping amount exerted a significant influence on the physicochemical properties of La-BDC-140-derived carbon materials. Figure 1 illustrates the preparation procedure of La-CNS_x_. Briefly, La-CNS_x_ was synthesized via pyrolysis using La-BDC-140 as the precursor with different thiourea doping amounts. The resulting carbon products exhibited slight variations in microstructure, indicating that the thiourea content played a role in regulating the material structure. To systematically investigate the effect of thiourea doping amount on the microstructural evolution of the materials, SEM observation and particle-size analysis were performed on five samples prepared with different doping ratios.

From the SEM images, La-CNS_1_ mainly exhibits an interlaced stacking morphology composed of numerous short rod-like and wire-like particles. The overall structure is relatively compact, although some interparticle voids can still be observed locally. At a low thiourea dosage, the La-BDC-140 precursor appears to partially retain its original one-dimensional nanowire-like morphology after pyrolysis, while the carbonized surface became noticeably rougher. This morphology suggests that the structural regulation effect of thiourea may be relatively limited at low dosage, resulting in a dense linear-stacked structure. The relatively underdeveloped pore structure may be associated with the limited amount of volatile species generated from thiourea decomposition during carbonization. The SEM morphology of La-CNS_2_ is similar to that of La-CNS_1_, still dominated by short rod-like or short fibrous structures. However, the overall morphology appears more uniform, and the distribution of the rod-like units is more regular. Compared with La-CNS_1_, the particle packing of La-CNS_2_ becomes slightly looser, with more visible interspaces and interconnected channels. These changes suggest that increasing thiourea content may contribute to the regulation of the carbon framework during pyrolysis, possibly through the introduction of S- and N-containing species and the release of volatile decomposition products. Such effects could favor the formation of additional defects and interparticle voids, leading to a more open microstructure than that of La-CNS_1_. La-CNS_3_ shows a more distinct morphological evolution. As observed in the image, this sample no longer presents a simple rod-stacked structure but instead displays an open architecture consisting of relatively thick short rods intertwined with pore channels. The pores become more evident, and the three-dimensional framework is more pronounced, showing typical features of a hierarchical porous structure. This result suggests that an appropriate thiourea dosage is beneficial for regulating the carbonization-derived microstructure of the La-BDC-140 precursor. The incorporation of S and N atoms may induce more defects and active sites in the carbon framework [27]. In addition, volatile species released during pyrolysis may contribute to pore development and the formation of a more open skeleton structure. However, since the intermediate pyrolysis process was not directly monitored, the proposed pore-forming effect should be regarded as a plausible explanation based on the observed SEM morphology and other structural characterizations. The morphology of La-CNS_4_ undergoes further transformation, changing markedly from the previous rod-like structure into larger sheet-like carbon architectures. These carbon sheets exhibit clear edges and relatively smooth surfaces, and they overlap with each other to form a loose layered stacking structure. This observation suggests that a higher thiourea dosage may further influence the structural evolution of the precursor during pyrolysis, leading to a gradual transition from rod-like structures to sheet-like carbon architectures. This may be related to enhanced heteroatom incorporation, release of volatile small molecules, and partial rearrangement of the carbonaceous framework during high-temperature treatment [28]. La-CNS_5_ mainly exhibits a wrinkled carbon nanosheet morphology. Compared with La-CNS_4_, the carbon sheets are flatter and more continuous overall, while still showing characteristic curling, overlapping, and localized wrinkles. Such morphological features are commonly observed in two-dimensional carbon-based materials and may be associated with structural relaxation and rearrangement during high-temperature carbonization. The predominance of nanosheet-like morphology in La-CNS_5_ suggests that excessive thiourea addition strongly affects the final carbon architecture, possibly through intensified heteroatom doping and volatile-species release. Overall, the morphology evolution from La-CNS_1_ to La-CNS_5_ indicates that thiourea dosage plays an important role in regulating the microstructure of La-BDC-derived carbon materials, although the detailed pyrolysis pathway requires further in situ or time-resolved characterization to be fully clarified [29,30]. Figure S2 EDS elemental mapping reveals that La-CNS_3_ is mainly composed of C, N, O, S, and La elements. Figure 1b–e presents the TEM images of the different La-CNS_x_ samples. High-resolution TEM images reveal distinct lattice fringes in all La-BDC-140-derived carbon samples, indicating a certain degree of structural ordering and crystallinity [31]. Specifically, lattice spacings of 0.352 and 0.383 nm were observed for La-CNS_1_, which can be assigned to the (100) plane of La_2_O_2_S and the (101) plane of the derived carbon, respectively, as shown in Figure 1b. For La-CNS_2_, the measured interplanar spacings were 0.355 and 0.374 nm, corresponding to the (100) plane of La_2_O_2_S and the (101) plane of the derived carbon, respectively (Figure 1c). In the cases of La-CNS_3_, La-CNS_4_, and La-CNS_5_, lattice spacings of approximately 0.375 and 0.694 nm were detected, which are attributed to the (101) plane of La_2_O_2_S and the (001) plane of the derived carbon, respectively (Figure 1d–f).

**Figure 2:**
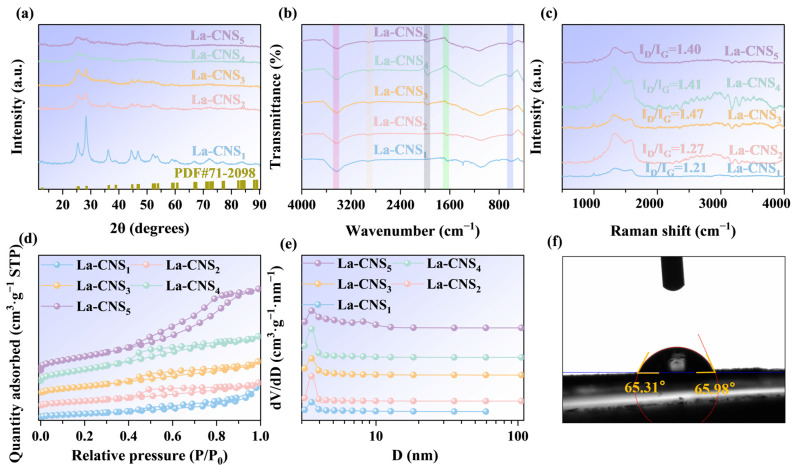
(**a**) XRD pattern, (**b**) FTIR spectra, (**c**) Raman spectra, (**d**) N_2_ adsorption–desorption isotherms, (**e**) BJH pore-size distribution profiles, (**f**) Contact angle test results of La-CNS_x_.

As shown in Figure 2a, La-CNS_1_ exhibits distinct crystalline diffraction peaks, and the characteristic peak positions are highly consistent with those of the standard PDF card PDF#71-2098, which is assigned to La_2_O_2_S [32]. In addition, phase analysis indicates the formation of g-C_3_N_4_, suggesting that the material retained a relatively high degree of crystallinity at a low thiourea doping level. With increasing thiourea content from La-CNS_1_ to La-CNS_5_, the diffraction peaks gradually weakened and broadened, eventually evolving into a broad diffuse peak resembling amorphous carbon in La-CNS_5_. This structural evolution indicates that excessive thiourea induces more intense framework reconstruction during high-temperature carbonization at 800 °C, progressively disrupting the long-range ordered crystalline structure and promoting the formation of highly exfoliated or disordered carbon nanostructures [33]. The FTIR spectra in Figure 2b further reveal the chemical bonding characteristics of the samples. All samples display a broad absorption band near 3400 cm^−1^, which can be attributed to the stretching vibrations of -OH or -NH groups. The absorption peak located at approximately 1630 cm^−1^ corresponds to the C=C or C=N stretching vibration in the sp^2^-hybridized carbon framework. Notably, with increasing thiourea dosage, slight changes are observed in the peak profiles within the range of 800~1500 cm^−1^, reflecting the successful incorporation of heteroatoms such as N and S into the carbon skeleton and the consequent modification of the local electronic environment [34]. Raman spectroscopy was further performed to quantitatively evaluate the defect density and structural ordering of the carbon materials. As shown in Figure 2c, all samples exhibit two typical bands at approximately 1350 cm^−1^ and 1580 cm^−1^, corresponding to the D band associated with disordered defects and the G band related to the graphitic sp^2^ carbon structure, respectively [35,36]. The I_D_/I_G_ ratio first increases and then decreases with increasing thiourea content, with La-CNS_3_ showing the highest I_D_/I_G_ value of 1.47. This result indicates that La-CNS_3_ possesses the highest defect density and a greater exposure of active sites [37]. The variation in I_D_/I_G_ further suggests that thiourea doping modulates the defect structure of the derived carbon materials. At the initial stage, thiourea incorporation introduces more structural defects, such as point defects and edge defects, thereby enhancing the D band and increasing the I_D_/I_G_ ratio. However, with further increases in thiourea content, thiourea-derived species may promote partial rearrangement or repair of the carbon lattice, leading to a decrease in the ID/IG ratio. As shown in Figure 2d, the N_2_ adsorption–desorption isotherms of the five La-CNS_x_ samples exhibit typical type-IV characteristics with H2-type hysteresis loops, indicating that the samples mainly possess disordered mesoporous structures in the range of 2~50 nm (Table 1). At low relative pressure values (P/P_0_ < 0.4), the adsorption capacity increases gradually, reflecting the initial contribution of micropores to N_2_ uptake. In the medium-to-high relative pressure region (0.4 < P/P_0_ < 0.9), the adsorption amount increases sharply and a pronounced hysteresis loop appears, which is associated with capillary condensation in mesopores. With increasing thiourea content, both the specific surface area and total pore volume of the five samples show an overall increasing trend [38]. These results indicate that, in this synthesis system, the pore structure and specific surface area of the materials can be effectively regulated by precisely controlling the amount of thiourea added. The average pore sizes of La-CNS_1_, La-CNS_2_, La-CNS_3_, La-CNS_4_, and La-CNS_5_ are 4.22, 2.53, 2.45, 2.21, and 3.34 nm, respectively. The pore-size distributions are mainly concentrated in the mesoporous range, suggesting relatively good pore uniformity (Figure 2e). Figure 2f and Figure S3a–d present the water contact-angle results of La-CNS_3_ and La-CNS_2_, La-CNS_3_, La-CNS_4_, and La-CNS_5_, respectively. The average contact angles from La-CNS_1_ to La-CNS_5_ are 76.42°, 71.57°, 65.65°, 60.03°, and 58.23°, respectively, all of which are below 90°, indicating that the obtained materials possess hydrophilic surfaces. As the sample number increases, the contact angle gradually decreases, suggesting a continuous improvement in surface wettability. Among these samples, La-CNS_3_ exhibits a moderate contact angle and favorable hydrophilicity [39].

As shown in Figure S4, combined with the XRD and other structural characterization results, the La-CNS_x_ samples are mainly composed of La_2_O_2_S and g-C_3_N_4_. The XPS survey spectra reveal the presence of La, O, S, C, and N elements on the sample surface, indicating that thiourea simultaneously served as sulfur and nitrogen sources during pyrolysis and participated in the formation of the La_2_O_2_S/g-C_3_N_4_ composite structure. In the high-resolution C 1s spectra, the peak located at approximately 284.8 eV can be assigned to C-C/C=C bonds or surface-adsorbed carbon. The peak at 286.4–286.8 eV corresponds to C-C species, while the signal at around 288.6~289.1 eV is mainly attributed to N-C=N groups in g-C_3_N_4_. In the N 1s spectra, the peak at 398.2–398.5 eV is associated with sp^2^-hybridized N in C=N-C, whereas the peak at 400.2~400.7 eV can be assigned to terminal C-N-H species [40]. These results further confirm the formation of a g-C_3_N_4_-type C-N framework. The O 1s spectra can be deconvoluted into three components, corresponding to lattice oxygen, surface oxygen species, and high-binding-energy oxygen-containing species. Specifically, the peak at 528.8~529.2 eV is attributed to La-O-related lattice oxygen in La_2_O_2_S, while the peak at 531.3~531.9 eV corresponds to surface-adsorbed oxygen, hydroxyl groups, or defect-related oxygen species. The peak at 532.9~533.7 eV can be assigned to adsorbed water and related species. In the S 2p spectra, the peaks at 160.5~161.0 eV and 161.7~162.2 eV are assigned to the S 2p_3/2_ and S 2p_1/2_ doublet of S^2−^ in La_2_O_2_S, respectively, demonstrating the successful incorporation of sulfur into the La-O-S lattice. The peak located at approximately 163.2~164.0 eV may originate from a small amount of C-S-C bonding, whereas the signals in the range of 166~169 eV are related to surface-oxidized sulfur species [41]. In the La 3d spectra, the main peaks at approximately 834~835 eV and 851~852 eV, together with their high-binding-energy satellite peaks, indicate that La predominantly exists in the La^3+^ oxidation state. Overall, these results demonstrate that, after pyrolysis at 800 °C, La-BDC-140 reacted with thiourea to form a composite material mainly consisting of La_2_O_2_S and g-C_3_N_4_ [42,43]. Thermogravimetric analysis was performed to evaluate the thermal stability of La-CNS_3_. As shown in Figure S5, the sample does not exhibit a pronounced continuous weight loss during heating; instead, it shows an initial weight gain followed by weight loss. In the low-temperature region, the sample mass remains nearly unchanged with only slight fluctuations, indicating a low content of surface-adsorbed water and volatile impurities. As the temperature increases to 200~850 °C, the sample mass gradually increases. This behavior is mainly attributed to the oxidation of S^2−^ species in La_2_O_2_S under an air atmosphere and the formation of La-O-S or sulfate-like intermediate species, suggesting an obvious oxidation-induced weight gain during heating. Upon further heating to the high-temperature region, the TG curve begins to decline, and a distinct weight-loss peak appears at 920.86 °C in the DTG curve. This peak corresponds to the thermal decomposition of oxidized sulfur species or sulfate-like species, accompanied by the release of SO_x_ gases, and may also involve further decomposition of the residual carbon–nitrogen framework. The mass loss at this stage is approximately 2.74%, demonstrating that the La_2_O_2_S/g-C_3_N_4_ composite possesses good high-temperature stability [44].

### 3.2. Electrochemical Analysis

**Figure 3:**
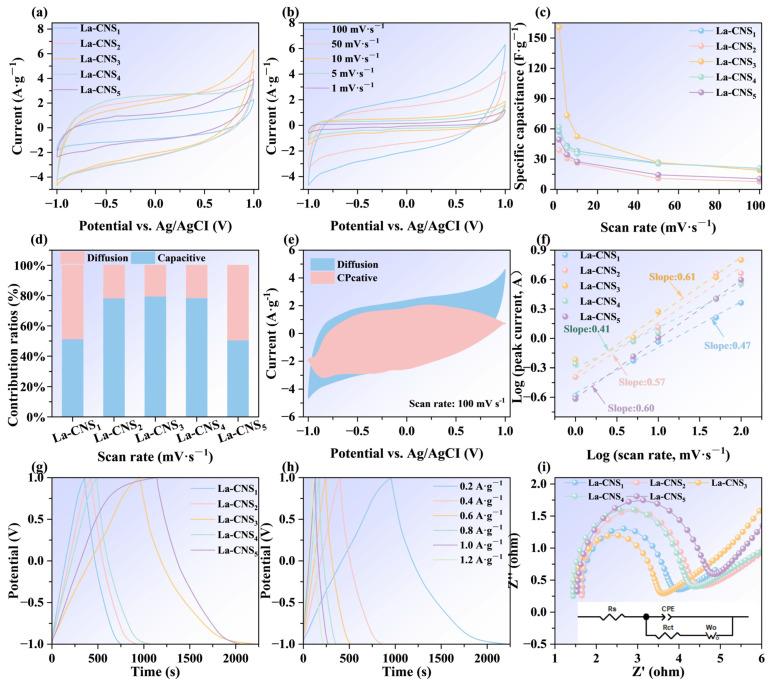
(**a**) CV curves of La-CNS_x_ at a scan rate of 100 mV·s^−1^, (**b**) CV curves of La-CNS_3_ at different scan rates (1, 5, 10, 50, and 100 mV·s^−1^), (**c**) specific capacitance of La-CNS_x_ at 1, 5, 10, 50, and 100 mV·s^−1^, (**d**) capacitive and diffusion-controlled contributions of La-CNS_x_ electrode at 100 mV·s^−1^, (**e**) capacitive and diffusion-controlled contributions of La-CNS_3_ at 100 mV·s^−1^, (**f**) power–law relationship between peak current and scan rate for La-CNS_x_, (**g**) GCD curves of La-CNS_x_ at a current density of 0.2 A·g^−1^, (**h**) GCD curves of La-CNS_3_ at various current densities (0.4, 0.6, 0.8, 1.0, and 1.2 A·g^−1^), and (**i**) EIS of La-CNS_x_ in a three-electrode system (inset: equivalent circuit diagram).

To systematically evaluate the electrochemical properties of La-CNS_x_ as CDI electrode materials, CV, GCD, and EIS measurements were conducted in a 1.0 M NaF electrolyte using a three-electrode system. As shown in Figure 3a, the CV curves of all La-CNS_x_ electrodes at a scan rate of 100 mV·s^−1^ display nearly rectangular shapes without obvious redox peaks, indicating favorable interfacial charge-transfer behavior and rapid ion-diffusion capability [45,46]. Among these samples, La-CNS_3_ exhibits the largest enclosed CV area, suggesting a higher charge-storage capacity and superior electrochemical response at the same scan rate, which is beneficial for enhancing ion electrosorption capacity during the CDI process. The CV behavior of La-CNS_3_ was further investigated at different scan rates. As presented in Figure 3b, when the scan rate increases from 1 to 100 mV·s^−1^, the CV curves still maintain a quasi-rectangular shape without significant distortion, demonstrating that La-CNS_3_ possesses good capacitance retention and rate capability under rapid charge–discharge conditions. The current response increases progressively with increasing scan rate, indicating that ion adsorption/desorption and charge accumulation can proceed rapidly on the electrode surface. Based on the CV curves recorded at 100 mV·s^−1^, La-CNS_3_ delivers the highest specific capacitance of 160.63 F·g^−1^, suggesting its superior ion-transport pathways and favorable electrode/electrolyte interfacial contact (Figure 3c). To further clarify the charge-storage mechanism of the La-CNS_x_ electrodes, the capacitive-controlled and diffusion-controlled contributions were distinguished. As shown in Figure 3d, La-CNS_3_ presents a relatively high capacitive contribution at a scan rate of 100 mV·s^−1^, indicating that its electrochemical process is not solely governed by ion diffusion within the pores but also involves pronounced surface capacitive behavior. Figure 3e further illustrates the capacitive contribution distribution of La-CNS_3_ at a scan rate of 100 mV·s^−1^. The capacitive-controlled region occupies the dominant portion, demonstrating that La-CNS_3_ can still achieve rapid surface charge storage at high scan rates. This feature is favorable for improving the ion removal efficiency and cycling response rate of CDI electrodes within a short operation time. In addition, according to the relationship between peak current and scan rate shown in Figure 3f, the b-values of all La-CNS_x_ samples fall within the range of 0.5~1.0, indicating that their charge-storage processes are jointly controlled by diffusion-controlled and capacitive-controlled behaviors [47]. Among them, La-CNS_3_ shows a b-value of approximately 0.61, which is higher than those of La-CNS_1_, La-CNS_2_, La-CNS_4_, and La-CNS_5_, suggesting a faster electrochemical response and a more prominent surface capacitive contribution. Overall, the CV curve area, specific capacitance variation, and kinetic analysis consistently demonstrate that La-CNS_3_ exhibits the best electrochemical performance among the La-CNS_x_ series. This superior performance may be attributed to the synergistic effects of its appropriate pore structure, abundant accessible active sites, and favorable electrolyte wettability. These results indicate that La-CNS_3_ is more conducive to rapid ion transport and effective ion adsorption within the electrode, making it a promising CDI electrode material in this series. To further assess the charge-storage capability of the La-CNS_x_ electrode materials, galvanostatic charge–discharge (GCD) measurements were performed. As shown in Figure 3g, the GCD curves of all La-CNS_x_ samples exhibit nearly symmetric triangular profiles at the same current density, indicating good reversibility during the charge–discharge process. This behavior suggests that the charge-storage process is mainly governed by electrochemical double-layer capacitance. Among the tested samples, La-CNS_3_ shows the longest charge–discharge duration. Figure 3h presents the GCD curves of La-CNS_3_ at different current densities. As the current density increases from 0.2 to 1.2 A·g^−1^, the charge–discharge time gradually decreases, which can be attributed to the shortened ion-diffusion and charge-accumulation time at high current densities. Under such conditions, some pore structures may not be fully accessible to electrolyte ions. Nevertheless, the GCD curves of La-CNS_3_ retain a well-defined triangular shape at different current densities, indicating favorable charge–discharge reversibility. The relatively long discharge time suggests that La-CNS_3_ can provide more effective charge-storage sites. Electrochemical impedance spectroscopy was further conducted to investigate the interfacial charge-transfer behavior of the La-CNS_x_ electrodes. As shown in Figure 3i, the Nyquist plots of all samples consist of a semicircle in the high-frequency region and an inclined line in the low-frequency region. The high-frequency semicircle is associated with the charge-transfer resistance at the electrode/electrolyte interface, whereas the low-frequency inclined line reflects the diffusion behavior of electrolyte ions within the electrode pores [48,49]. La-CNS_3_ exhibits a smaller semicircle radius in the high-frequency region, indicating lower interfacial charge-transfer resistance. Meanwhile, its more pronounced slope in the low-frequency region suggests faster ion diffusion inside the electrode.

### 3.3. Defluorination Performance

**Figure 4:**
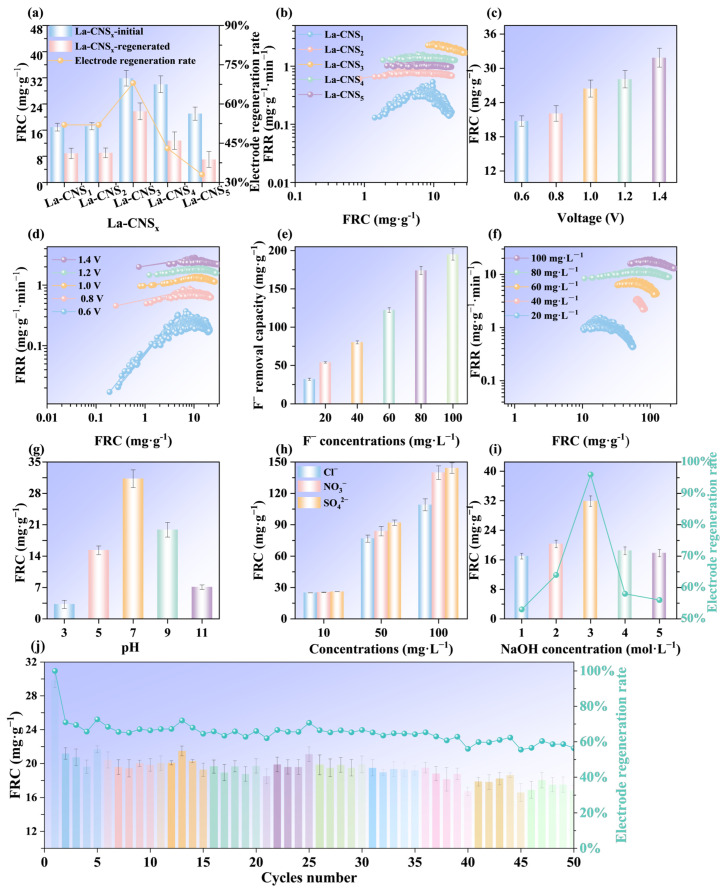
(**a**) FRC of different La-CNS_x_ samples and (**b**) Ragone plot; (**c**) FRC curves of La-CNS_3_ at different voltages and (**d**) Ragone plot; (**e**) FRC of La-CNS_3_ at different F^−^ concentrations as the reaction proceeds and (**f**) Ragone plot; FRC of La-CNS_3_ under different conditions: (**g**) pH, (**h**) coexisting ions, (**i**) NaOH concentration and (**j**) Regeneration performance over 50 cycles driven solely by an electric field.

The prepared La-CNS_x_ materials were used as the anode, while AC was employed as the cathode to construct a CDI system for evaluating fluoride removal performance. Except for the concentration-dependent experiments, a 10 mg·L^−1^ F^−^ solution was used as the target solution. The FRC of the electrodes was calculated according to the variation in effluent F^−^ concentration with operation time. Both the adsorption under forward voltage and the desorption under reverse voltage were carried out for 120 min to ensure consistent experimental conditions. Figure 4a compares the initial FRC, the FRC after regeneration under reverse voltage, and the electrode regeneration rate of different La-CNS_x_ electrodes at 1.4 V. The fluoride removal performance of the La-CNS_x_ electrodes first increases and then decreases with changing thiourea dosage. Among them, La-CNS_3_ exhibits the highest initial FRC of approximately 31.86 mg·g^−1^ at an initial F^−^ concentration of 10 mg·L^−1^, which is higher than those of most other samples. Although La-CNS_1_ shows an initial FRC close to that of La-CNS_3_, its regenerated FRC and electrode regeneration efficiency are much lower. In contrast, La-CNS_3_ still maintains an FRC of approximately 22 mg·g^−1^ after regeneration, with an electrode regeneration rate of about 70%, indicating superior reversible adsorption/desorption behavior. These results suggest that an appropriate thiourea content is beneficial for regulating the formation of the La_2_O_2_S/g-C_3_N_4_ composite structure, optimizing the pore architecture and surface active-site distribution, and thereby enhancing F^−^ capture. According to the SEM and electrochemical characterization results, excessive thiourea may lead to aggregation of active components, blockage of pore channels, or reduced conductivity, which decreases the number of accessible adsorption sites and consequently weakens the defluoridation performance of the electrode. The Ragone plot reflects both FRC and FRR and is therefore an important indicator for evaluating the overall defluoridation performance of CDI electrodes. Figure 4b presents the Ragone plots of different La-CNS_x_ electrodes at 1.4 V. The curve of La-CNS_1_ is located in the lower-left region, indicating relatively low FRC and FRR. With increasing thiourea content, the curves of La-CNS_2_, La-CNS_4_, and La-CNS_5_ shift gradually toward the upper-right region, suggesting improved fluoride removal capacity and rate. Notably, La-CNS_3_ is positioned at the upper-rightmost region, demonstrating that it possesses both a high fluoride removal capacity and a rapid fluoride removal rate. Therefore, La-CNS_3_ exhibits the best overall defluoridation performance and was selected as the representative electrode material for subsequent voltage- and concentration-dependent experiments. To further optimize the CDI operating conditions, the effect of applied voltage on the defluoridation performance of La-CNS3 was investigated. As shown in Figure 4c, when the voltage increases from 0.6 to 1.4 V, the FRC gradually increases from approximately 20.75 to 31.86 mg·g^−1^ at an initial F^−^ concentration of 10 mg·L^−1^. Based on an electrode material mass of 15 mg, the removed fluoride mass was calculated to be 0.478 mg. The total charge passed through the CDI cell, obtained by integrating the current–time curve, was 2.777 C. The theoretical charge required for the removed F^−^ was 2.43 C, resulting in a charge efficiency of 87.4%. In addition, the electrical energy input obtained by integrating the voltage-current-time profile was 1.080 mWh, corresponding to a specific energy consumption of 2.26 kWh·kg^−1^ F^−^ removed under 1.4 V. This indicates that increasing the applied voltage can strengthen the interfacial electric field, promote the migration and enrichment of F^−^ toward the electrode surface, and thereby enhance the fluoride removal capacity. The Ragone plots under different voltages in Figure 4d further confirm that the curves shift toward the upper-right region with increasing voltage, indicating simultaneous improvements in both FRC and FRR. In particular, La-CNS_3_ exhibits the highest fluoride removal capacity and a relatively fast removal rate at 1.4 V. Therefore, 1.4 V can be regarded as the optimal operating voltage for this CDI system. Figure 4e shows the influence of initial F^−^ concentration on the fluoride removal capacity of the La-CNS_3_ electrode. As the initial F^−^ concentration increases from 10 to 100 mg·L^−1^, the FRC gradually increases and reaches 195 mg·g^−1^ at 100 mg·L^−1^. This value is significantly higher than those of previously reported defluoridation materials [50,51,52,53] summarized in Table S1, highlighting the superior fluoride removal performance of La-CNS_3_. This phenomenon can be mainly attributed to the increased number of F^−^ ions in the high-concentration solution and the enhanced concentration gradient, which provide a stronger mass-transfer driving force for F^−^ diffusion and intercalation toward the electrode surface, thereby significantly improving the fluoride removal capacity per unit mass of electrode [54]. The Ragone plots in Figure 4f also show that the curves shift upward and to the right as the initial F^−^ concentration increases, indicating that La-CNS_3_ exhibits not only higher FRC but also improved FRR under high-concentration conditions. These results demonstrate that the La-CNS_3_ electrode possesses favorable defluoridation capability over a wide range of F^−^ concentrations and is particularly suitable for the rapid electrochemical treatment of high-fluoride wastewater. To evaluate the applicability and regeneration behavior of the La-CNS_3_ electrode under different water-quality conditions, the effects of initial pH, coexisting anions, and NaOH concentration on its CDI-based defluoridation performance were systematically investigated [55]. As shown in Figure 4g, the solution pH has a pronounced influence on the fluoride removal capacity of La-CNS_3_ [56]. The FRC increases markedly as the pH rises from 3 to 7, reaching its maximum under neutral conditions. However, when the pH further increases to 9 and 11, the FRC gradually decreases. Under acidic conditions, the high concentration of H^+^ can readily combine with F^−^ to form HF, thereby reducing the effective concentration of free F^−^ in solution. Meanwhile, H^+^ may also compete with F^−^ for active sites on the electrode surface, suppressing the electrosorption and coordination capture of fluoride. In alkaline media, abundant OH^−^ competes with F^−^ for adsorption sites and may preferentially occupy La-related active centers, weakening the La-F interaction and consequently decreasing the defluoridation capacity. The coexisting-anion experiments show that the FRC increases with increasing concentrations of both F^−^ and the coexisting anions (Cl^−^, NO_3_^−^, and SO_4_^2−^). It should be noted that, in these experiments, the initial F^−^ concentration was set equal to the concentration of the coexisting anion in order to simulate complex fluoride-containing water matrices with comparable levels of fluoride and competing ions. At the same concentration, the FRC obtained in the presence of SO_4_^2−^ is higher than that obtained with Cl^−^ and NO_3_^−^, indicating that the La-CNS_3_ electrode maintains good anti-interference capability and fluoride selectivity in sulfate-containing complex water matrices (Figure 4h). Significant emphasis is placed on electric-field-driven regeneration, which is achieved solely by reverse voltage in this study. For comparison, Figure 4i includes regeneration data from a previous study using NaOH-assisted desorption. As the NaOH concentration decreases from 0.15 to 0.05 mol·L^−1^, both the regenerated FRC and F^−^ release ratio first increase and then decrease, reaching their optimum values at 0.1 mol·L^−1^ NaOH, where the electrode regeneration rate approaches 95%. This result suggests that an appropriate NaOH concentration can effectively promote the desorption of adsorbed F^−^ and restore the active sites of the electrode. However, excessively high or low NaOH concentrations both lead to decreased FRC and electrode regeneration rate. This may be because a high-concentration alkaline solution can damage the electrode surface structure, whereas insufficient alkalinity may fail to completely detach F^−^ from the occupied active sites, thereby weakening the regeneration performance of the electrode [57]. To further assess the long-term operational stability of the La-CNS_3_ electrode during CDI-based defluoridation, 50 consecutive adsorption–regeneration cycles were performed at an applied adsorption/desorption voltage of ±1.4 V, with both adsorption and desorption periods set to 1 h. As shown in the figure, during the first 30 cycles, the FRC remained nearly stable at approximately 20 mg·g^−1^ without obvious attenuation, indicating that La-CNS_3_ maintained a high F^−^ capture capability during repeated electrosorption/desorption processes. Meanwhile, the FRR consistently remained close to or above 65%, suggesting that electric-field-driven regeneration effectively promoted the release of adsorbed F^−^ and enabled sufficient recovery of the surface active sites. With further cycling, both the FRC and electrode regeneration efficiency decreased to some extent. In particular, after 40 cycles, the FRC declined to approximately 16~19 mg·g^−1^, and the electrode regeneration efficiency, defined as the ratio of desorbed F^−^ under reverse voltage to adsorbed F^−^ under forward voltage, decreased to approximately 55%. After approximately 40 adsorption–desorption cycles, both the FRC and electrode regeneration efficiency decreased noticeably. To further investigate the origin of this decline, SEM and EDS analyses were performed on the La-CNS_3_ electrode after 40 cycles (Figure S6). The post-cycling SEM images show that the electrode surface is partially covered by fluoride-containing deposits, while the EDS results confirm the presence of F on the cycled electrode. This suggests that part of the adsorbed F^−^ species cannot be completely removed during reverse-voltage regeneration. Therefore, the gradual decrease in cycling performance may be attributed to the combined effects of irreversible or strongly retained La-F species, partial blockage of pores/channels by accumulated fluoride-containing species, and possible local structural changes during repeated adsorption–desorption cycles. Groundwater from Village 15, Duletbage Township, Kashi City, Kashi Prefecture, Xinjiang, was collected as the actual water sample in this study. Basic water quality parameters, including F^−^ concentration and pH, were measured and analyzed, and the results are presented in Table S2. Since the F^−^ concentration in the raw water was relatively low and could not meet the experimental requirements for subsequent defluoridation performance evaluation, the actual groundwater sample was spiked according to the preparation method used for the simulated fluoride-containing water in the preliminary experiments. The initial F^−^ concentration was uniformly adjusted to 10 mg·L^−1^. As shown in Figure S7, the F^−^ concentration changed slowly over time under the non-electrified condition. In contrast, under an applied voltage of 1.4 V, the effluent F^−^ concentration decreased to below 1.0 mg·L^−1^ after 120 min, meeting the requirement specified in WHO *Standards for Drinking Water Quality*.

Kinetic analysis of the F^−^ removal rate is helpful for understanding the ion-capture rate of the electrode and its response characteristics over the operation time. Therefore, the electrically driven defluoridation behavior of La-CNS_3_ was fitted and analyzed at different initial fluoride concentrations. The experimental data were processed using the pseudo-first-order kinetic model (Equation (4)) and the pseudo-second-order kinetic model (Equation (5)) to evaluate their applicability to fluoride removal under CDI conditions.

**Figure 5:**
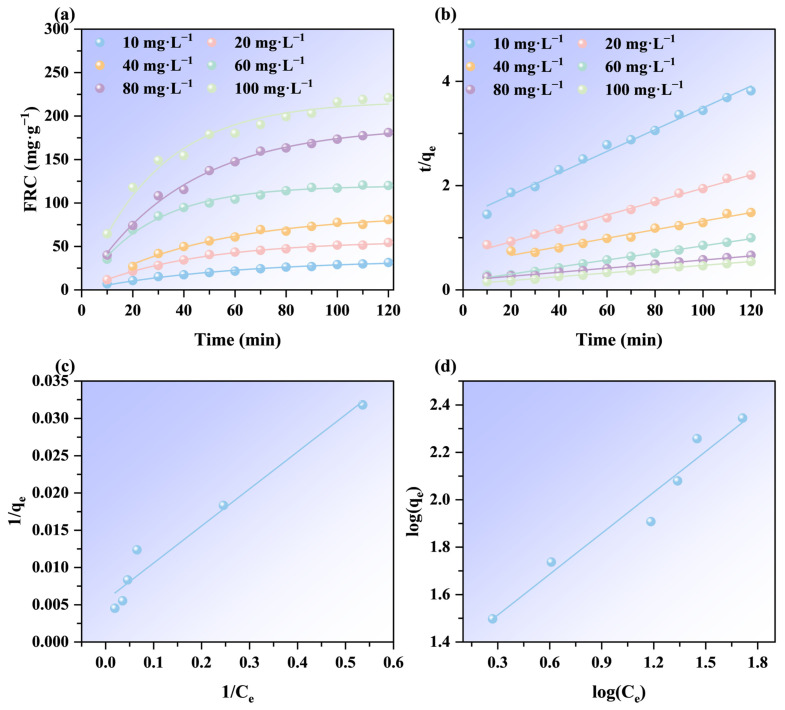
(**a**) pseudo-first-order kinetic model, (**b**) pseudo-second-order kinetic model, (**c**) Langmuir adsorption isotherm model, and (**d**) Freundlich adsorption isotherm model of La-CNS_3_.

As shown in Figure 5a,b, both kinetic models can describe the adsorption kinetic data of La-CNS_3_ reasonably well. However, the pseudo-second-order model provides a better fitting performance, as evidenced by its higher correlation coefficient (R^2^) and fitting parameters that are closer to the experimental values (Table 2). In addition, the equilibrium adsorption capacity calculated from this model agrees well with the experimental results. These findings indicate that the F^−^ adsorption process is mainly governed by chemical interactions, and the adsorption rate is closely related to the number of available active sites remaining on the electrode surface. To further analyze the equilibrium adsorption behavior, the experimental data were fitted using nonlinear isotherm models, including the Langmuir model (Equation (6)) and the Freundlich empirical model (Equation (7)). The corresponding fitting results are summarized in Table 3 and presented in Figure 5c,d. The results show that the Langmuir model exhibits a better correlation than the Freundlich model, indicating that F^−^ enrichment on the La-CNS_3_ surface is more consistent with monolayer adsorption. This suggests that the adsorption sites are relatively independent, with each active site primarily binding one fluoride ion [58,59,60,61]. These results further demonstrate that the active centers responsible for F^−^ binding on the La-CNS_3_ surface are relatively uniform and that the defluoridation process is dominated by specific chemical interactions.

### 3.4. Defluorination Mechanism of La-CNS_3_

**Figure 6:**
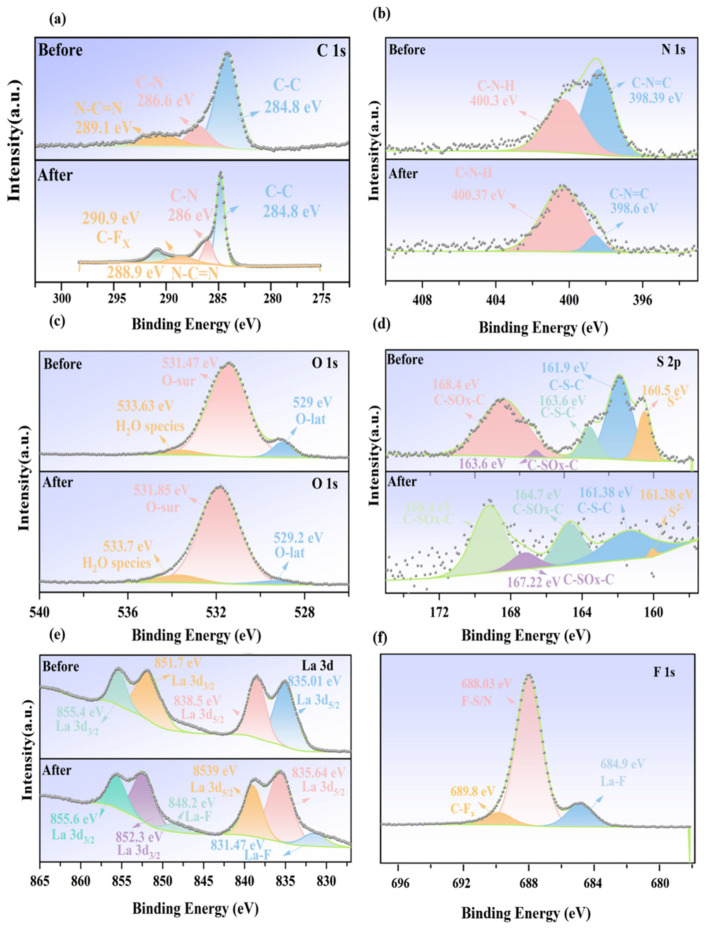
XPS spectra of La-CNS_3_ before and after defluoridation: (**a**) C 1s, (**b**) N 1s, (**c**) O 1, (**d**) S 2p, (**e**) La 3d and (**f**) F 1s.

Figure 6 presents the high-resolution XPS spectra of C 1s, N 1s, O 1s, S 2p, La 3d, and F 1s for La-CNS_3_ before and after fluoride removal. After F^−^ adsorption, the binding energies and peak profiles of these elements change to varying degrees, suggesting pronounced interactions between F^−^ and the surface active sites of La-CNS_3_ rather than simple physical adsorption. For the C 1s spectrum (Figure 6a), the pristine La-CNS_3_ sample can be deconvoluted into three peaks located at approximately 284.8, 286.6, and 289.1 eV, corresponding to C-C/C=C, C-N, and N-C=N species, respectively [62]. These peaks confirm the presence of a conjugated carbon–nitrogen framework derived from g-C_3_N_4_. After fluoride adsorption, the C-C peak remains nearly unchanged at around 284.8 eV, suggesting that the carbon framework is generally stable during the adsorption process. In contrast, the C-N peak shifts to approximately 286.0 eV, while the N-C=N peak shifts to around 288.9 eV, indicating that the electronic environment around nitrogen-containing carbon species is affected by F^−^ adsorption. Notably, a new high-binding-energy peak appears at approximately 290.9 eV after fluoride removal, which can be assigned to C-F_x_ or surface fluorinated carbon species. The appearance of this peak suggests that a small fraction of fluorine species may interact with edge defects or carbon-containing functional groups in the carbon framework. However, its relatively small peak area indicates that the carbon skeleton is not the dominant active site for F^−^ immobilization [63]. In the N 1s spectrum, the peaks located at 398.39 and 400.3 eV before fluoride adsorption are assigned to C-N=C and C-N-H species, respectively. After fluoride adsorption, these two peaks shift slightly to higher binding energies of 398.6 and 400.37 eV, respectively. This positive shift indicates a decrease in electron density around the N sites after F^−^ adsorption, which may be associated with electrostatic interactions between F^−^ and protonated nitrogen sites (Figure 6b). Therefore, the nitrogen-containing functional groups in g-C_3_N_4_ may serve as auxiliary adsorption sites for F^−^ enrichment, while also helping to anchor La active centers and regulate their electronic structure. Before fluoride adsorption, the O 1s spectrum can be fitted into three peaks at 529.0, 531.47, and 533.63 eV, corresponding to lattice oxygen, surface hydroxyl/adsorbed oxygen species, and adsorbed water, respectively. After fluoride adsorption, the lattice oxygen peak shifts to 529.2 eV, the surface oxygen peak shifts to 531.85 eV, and the adsorbed water peak shifts to 533.7 eV. In particular, the obvious shift in the surface hydroxyl-related oxygen peak indicates that La-OH, La-O, or surface-adsorbed oxygen species are involved in F^−^ binding [59,64]. This result suggests that F^−^ may partially replace surface hydroxyl groups or water ligands through ligand exchange and coordinate with La centers to form La-F species. (Figure 6c) [65]. In the S 2p spectrum, changes in both peak positions and peak areas are observed after fluoride adsorption, indicating that the electronic structure around S-doped sites in La_2_O_2_S is affected by F^−^ adsorption (Figure 6d). Meanwhile, the La 3d peaks show obvious shifts after defluoridation, accompanied by the emergence of La-F-related components, demonstrating strong interactions between F^−^ and La^3+^ centers. Because F^−^ has high electronegativity, the formation of La-F bonds reduces the electron density around La, thereby causing changes in the La 3d binding energy (Figure 6e). After fluoride adsorption, the F 1s spectrum can be deconvoluted into three peaks at approximately 684.9, 688.03, and 689.8 eV. Among them, the peak at 684.9 eV can be attributed to La-F species. Combined with the changes observed in the La 3d and O 1s spectra, these results support that La^3+^ active sites play an important role in F^−^ capture through coordination and possible ligand exchange on the surface of La_2_O_2_S (Figure 6f) [66]. The desorption results further indicate that, compared with the La_2_O_2_S electrode material discussed in the previous chapter, the La_2_O_2_S/g-C_3_N_4_ composite can achieve partial F^−^ release under voltage-driven conditions. This improvement may be related to the synergistic regulation of S^2−^ and g-C_3_N_4_. The introduction of S^2−^ into La_2_O_2_S may create a mixed La-O-S coordination environment around La sites, which is expected to improve interfacial polarization and charge redistribution. This may allow the applied reverse voltage to more effectively modulate the charge distribution at the material/solution interface. During the desorption stage, F^−^ weakly adsorbed near nitrogen-containing sites in g-C_3_N_4_, S-regulated interfaces, and surface defect sites may be preferentially released. Meanwhile, under the combined effect of the reverse electric field and OH^−^ competition, part of the La-F species may undergo reverse ligand exchange, regenerating La-OH sites and releasing F^−^ [57]. The fluoride removal reaction process can be expressed as follows [67]:

(10) $$La^{3+}\;+\;H_{2}O\;⇄\;La\mathrm{-O}H\;+\;H^{+};\;La\mathrm{-O}H\;+\;F^{-}\to La\mathrm{-F}\;+\;OH^{-}$$

(11) $$La\mathrm{-O}H\;+\;H^{+}\;⇄\;{La\mathrm{-O}H}_{2}^{+};\;{La\mathrm{-O}H}_{2}^{+}+F^{-}\to La\mathrm{-F}+H_{2}O$$

(12) $${C\mathrm{-N}H}^{+}+F^{-}\to {C\mathrm{-N}H}^{+}\ldots F$$

The F^−^ desorption process can be described by the following reactions:

(13) $$La\mathrm{-F}\;+\;{OH}^{-}\underset{\mathrm{Reverse}\;\mathrm{voltage}}{\to }La\mathrm{-O}H\;+{\;F}^{-}$$

(14) $${C\mathrm{-N}H}^{+}\ldots F\underset{\mathrm{Reverse}\;\mathrm{voltage}}{\to }{C\mathrm{-N}H}^{+}+F^{-}$$

**Figure 7:**
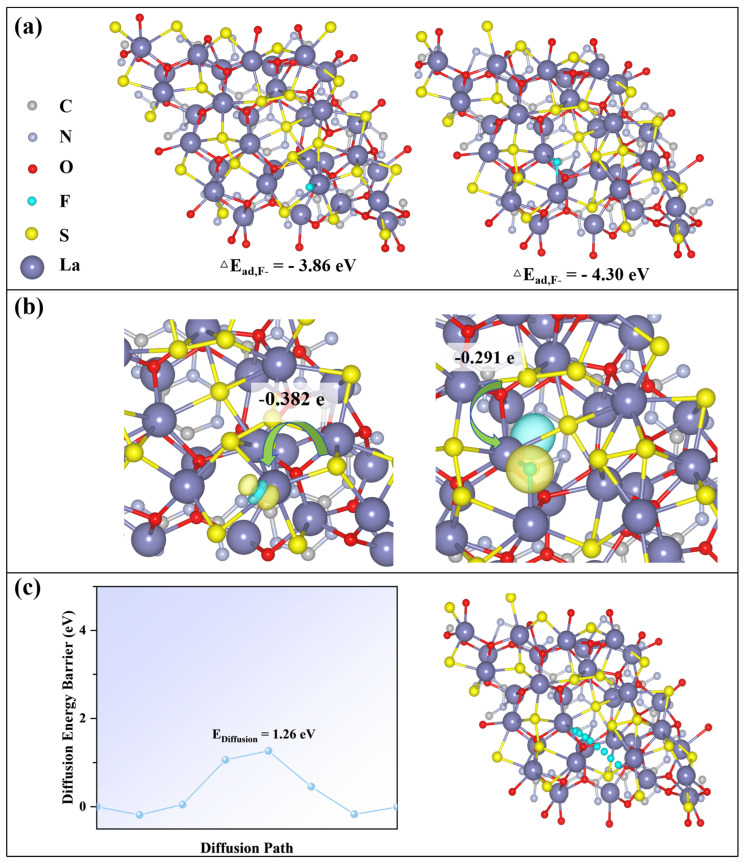
(**a**) Adsorption models of La-CNS_3_ at different sites; (**b**) charge density difference during the fluoride removal process; (**c**) migration energy barrier of F^−^ in La-CNS_3_ based on DFT calculations.

To further elucidate the reason why partial F^−^ desorption can be achieved on La-CNS_3_, density functional theory (DFT) calculations were performed on the optimized La_2_O_2_S/g-C_3_N_4_ theoretical model. All DFT calculations were performed using the CP2K 2025.2 program. The exchange–correlation potential was described by the Perdew–Burke–Ernzerhof (PBE) generalized gradient approximation (GGA), with Grimme‘s DFT-D3(BJ) dispersion correction [68,69]. The bulk La_2_O_2_S/g-C_3_N_4_ model was obtained from the Materials Project database. CP2K input files were automatically generated using Multiwfn, which was also used for post-processing analysis, charge-density evaluation, and migration energy barrier calculations [70]. Spin-polarized calculations were carried out throughout. GTH pseudopotentials were employed to describe the ion–electron interaction. The DZVP-MOLOPT-SR-GTH basis set was used for geometry optimizations and CI-NEB calculations, while the TZV2P-MOLOPT-SR-GTH basis set was used for single-point energy calculations. A Gaussian–plane-wave (GPW) scheme was adopted with a CUTOFF of 400 Ry and REL_CUTOFF of 55 Ry. The Brillouin zone was sampled only at the Γ-point. The electronic self-consistent field (SCF) convergence criterion was set to 1.0 × 10^−6^ Hartree. The geometry optimization convergence criteria were: MAX_FORCE < 4.5 × 10^−4^ Hartree/Bohr, RMS_FORCE < 3.0 × 10^−4^ Hartree/Bohr, MAX_DR < 3.0 × 10^−3^ Bohr, and RMS_DR < 1.5 × 10^−3^ Bohr. The Climbing-Image Nudged Elastic Band (CI-NEB) method was used to calculate the diffusion barrier, with 6 images inserted between the optimized initial and final states. All wavefunction analyses were carried out using Multiwfn 3.8 Dev. The optimized structures and diffusion paths were visualized using VESTA [71,72]. Figure 7a shows the adsorption energies of F^−^ at different sites. The variation in adsorption energy suggests that F^−^ binding strength is site-dependent, which may contribute to the partial desorption behavior under voltage-driven conditions. Figure 7b presents the differential charge density maps for F^−^ adsorption at different sites, where the yellow regions represent charge accumulation and the green regions indicate charge depletion. The charge transfer amounts of the two adsorption configurations are −0.382e and −0.291e, respectively, suggesting obvious electron redistribution between the material surface and F^−^ during adsorption [73]. Although the configuration with an adsorption energy of −3.86 eV shows a slightly weaker adsorption strength than that with −4.30 eV, it exhibits a larger charge transfer, indicating stronger interfacial charge response and polarization at this site. This suggests that the adsorption stability of F^−^ is not solely determined by the amount of charge transfer, but is also jointly affected by La coordination strength and interfacial regulation from the La_2_O_2_S/g-C_3_N_4_ structure. A relatively lower adsorption energy indicates weaker F^−^ binding and may facilitate F^−^ release during the desorption process, which is essential for efficient electrosorption–desorption cycling. This interpretation is consistent with the migration energy barrier analysis shown in Figure 7c, suggesting that La_2_O_2_S/g-C_3_N_4_ may facilitate F^−^ surface migration and active-site renewal, thereby facilitating active-site renewal and overall mass transfer. Overall, these results suggest that La_2_O_2_S/g-C_3_N_4_ may provide a favorable balance between F^−^ adsorption strength and desorption feasibility. This balance is critical for maintaining high defluoridation capacity, reversible ion release, long-term stability, and energy efficiency during CDI operation.

## 4. Discussion

This study employed La-BDC-140 as the precursor to develop an S, N-co-regulated La_2_O_2_S/g-C_3_N_4_-derived carbon electrode, denoted as La-CNS_3_, through a thiourea-assisted carbonization strategy. This design enables La-based defluoridation materials to move beyond conventional strong adsorption toward the integration of efficient fluoride capture and electric-field-driven regeneration. The results indicate that an appropriate amount of thiourea can synergistically regulate the phase composition, pore structure, defect density, and surface electronic environment of the material, thereby endowing La-CNS_3_ with abundant defects, a favorable mesoporous architecture, enhanced hydrophilicity, and rapid charge-transfer capability. As a result, La-CNS_3_ exhibits superior defluoridation performance in the CDI system. At 1.4 V, La-CNS_3_ delivers an initial fluoride removal capacity of 31.86 mg·g^−1^ for a 10 mg·L^−1^ F^−^ solution and maintains a capacity of approximately 22 mg·g^−1^ after regeneration. When the initial F^−^ concentration is increased to 100 mg·L^−1^, the fluoride removal capacity reaches 195 mg·g^−1^, indicating its high fluoride uptake capacity and adaptability to different fluoride concentrations.

More importantly, partial F^−^ desorption can be achieved under a reverse electric field without the use of additional chemical regenerants, and the electrode retains favorable defluoridation performance after 50 adsorption–regeneration cycles. Nevertheless, the 50-cycle test should be regarded as a preliminary cycling-stability evaluation, and longer-term cycling studies are still needed to fully assess its practical durability. Based on the current-voltage profiles, the charge efficiency and specific energy consumption of La-CNS_3_ were calculated to be 87.4% and 2.26 kWh·kg^−1^ F^−^ removed, respectively, further indicating its reasonable charge utilization and energy efficiency under the tested conditions.

Ex situ XPS analysis and DFT calculations suggest that fluoride removal by La-CNS_3_ may be jointly governed by La-F coordination, surface hydroxyl/water ligand exchange, nitrogen-site-assisted fluoride enrichment, and interfacial charge redistribution. In particular, the La_2_O_2_S/g-C_3_N_4_ composite structure provides moderate F^−^ adsorption energy and a reduced migration energy barrier, which may help balance fluoride adsorption strength and desorption reversibility. It should be noted that the proposed reversible La-F bond formation/cleavage mechanism is mainly supported by ex situ XPS, electrochemical adsorption/desorption behavior, cycling performance, and DFT calculations, rather than direct in situ spectroscopic observation. Therefore, future in situ or operando Raman, FTIR, or X-ray absorption spectroscopic studies are needed to further verify the dynamic evolution of La-F coordination under applied potential. Overall, this study provides a feasible design strategy and theoretical basis for developing efficient, selective, and regenerable rare-earth-based CDI electrodes for fluoride removal.

## Figures and Tables

**Table 1:** The specific surface area, porosity and pore size parameter.

Sample	SBET (cm^2^·g^−1^)	Pore Size (nm)	Vtotal (cm^3^·g^−1^)
La-CNS_1_	11.58	4.22	2.66
La-CNS_2_	16.15	2.53	3.71
La-CNS_3_	23.31	2.45	5.36
La-CNS_4_	35.91	2.21	8.25
La-CNS_5_	40.38	3.34	9.28

**Table 2:** The specific parameter values of pseudo-first order model and pseudo-second order model.

*C*_0_ (mg·L^−1^)	Models	*K*_1_ (10^−3^)	q_e_ (mg·g^−1^)	*R* ^2^	Models	*K*_2_ (10^−3^)	q_e_ (mg·g^−1^)	*R* ^2^
10.00	Pseud first-order	0.017	34.85	0.9906	Pseudo second-order	0.31	47.92	0.9871
20.00		0.024	56.45	0.9655		0.25	78.00	0.9705
40.00		0.021	86.58	0.9646		0.13	122.10	0.9732
60.00		0.039	119.83	0.9884		0.27	147.71	0.9937
80.00		0.026	188.64	0.9848		0.08	199.07	0.9885
100.00		0.035	217.25	0.9974		0.12	274.73	0.9950

**Table 3:** Parameters of the fluoride adsorption isotherm models for La-CNS_3_.

T	Langmuir Model			Freundlich Model		
	*q_m_* (mg·g^−1^)	*K_L_* (L·mg^−1^)	*R* ^2^	*K_f_* (L·mg^−1^)	1/*n*	*R* ^2^
298 K	177.94	0.11	0.9616	21.88	0.54	0.94

## Data Availability

The original contributions presented in this study are included in the article/Supplementary Materials. Further inquiries can be directed to the corresponding authors.

## Supplementary Materials

The following supporting information can be downloaded at: https://www.mdpi.com/article/10.3390/ma19122556/s1, Figure S1: (a) The structure diagram of CDI cell and (b) experimental setup used in this work; Figure S2: EDS elemental mapping of LZC_3_: (a) electron image, (b) C, (c) N, (d) O, (e) S and (f) La; Figure S3: Contact angle test images of La-CNS_x_: (a) La-CNS_1_, (b) La-CNS_2_, (c) La-CNS_4_, (d) La-CNS_5_; Figure S4: XPS spectra of La-CNS_x_: (a) O 1s spectra; (b) S 2p spectra; and (c) La 3d spectra; Figure S5: TG and DTG curves of La-CNS_3_; Figure S6: SEM images and EDS mapping of La-CNS_3_ after 40 cycles.; Figure S7: Variation of F^−^ concentration with time during static adsorption and under an applied voltage of 1.4 V at an initial F^−^ concentration of 10 mg·L^−1^; Table S1: Comparison of the fluoride adsorption capacity and regeneration efficiency of La-CNS_3_ with recently reported CDI-derived carbon materials; Table S2: Parameters of the collected groundwater samples.
