# Supplementary material for: From Strong Fluoride Binding to Reversible Electrodesorption: S, N-Regulated La-MOF-Derived Carbon Electrodes for Capacitive Deionization Defluoridation
Source: Materials (Basel). 2026 Jun 12;19(12):2556. doi: 10.3390/ma19122556 (PMC13303663; doi:10.3390/ma19122556)
Supplement: Supplementary file 1 [file materials-19-02556-s001.zip › materials-4355763-supplementary.pdf]

Supplement Information

From Strong Fluoride Binding to Reversible Electrodesorption: S, N-Regulated La-MOF-Derived Carbon Electrodes for Capacitive Deionization Defluoridation

Xue Yang [1,2], Shirong Yang [1], Dongbao Song [1], Hongtao Zhang [1], Junfeng Li [1,*] and Pu Wang [1,*]

[1] College of Water Conservancy and Architectural Engineering, Shihezi University, Shihezi 832000, China; yangxue@stu.shzu.edu.cn (X.Y.); yangshirong@stu.shzu.edu.cn (S.Y.); songdongbao94@163.com (D.S.); hongtao.zhang@shzu.edu.cn (H.Z.)

[2] Water Resources and Water Environment Engineering Technology Center, Xinjiang Key Laboratory of Synthesis and Application of Carbon Nanomaterials, School of Civil Engineering, Kashi University, Kashi 844000, China

* Correspondence: lijunfeng@shzu.edu.cn (J.L.); wpu2120@cqu.edu.cn (P.W.); Tel.: +86 152 9992 1362 (J.L.)

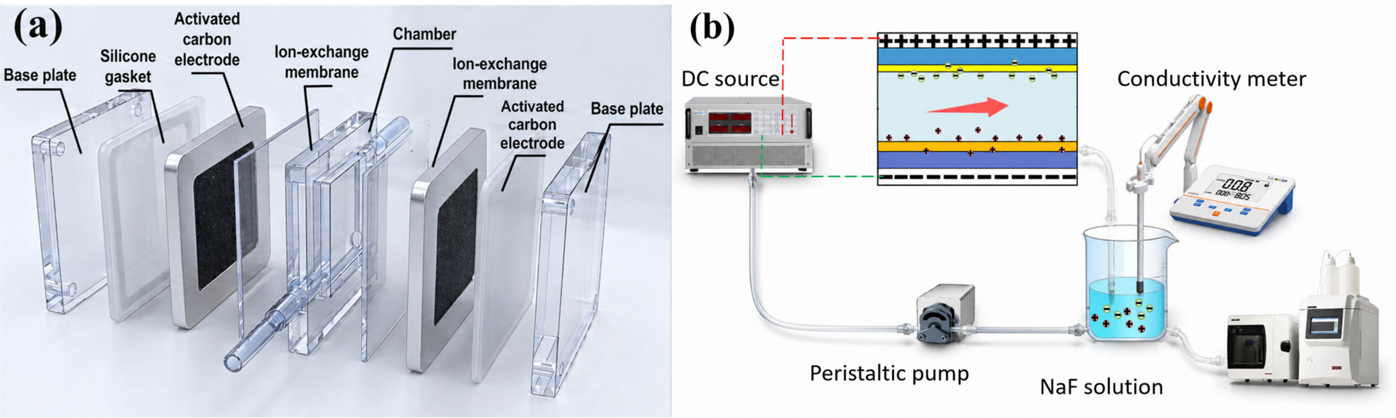

Figure S1. (a) The structure diagram of CDI cell and (b) experimental setup used in this work.

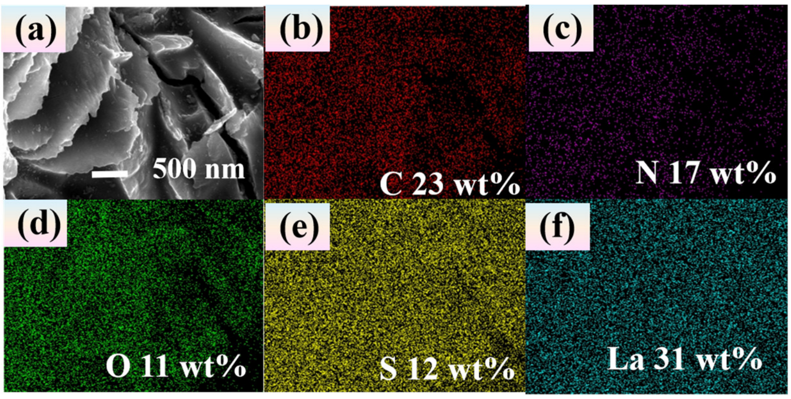

Figure S2. EDS elemental mapping of LZC_3: (a) electron image, (b) C, (c) N, (d) O, (e) S and (f) La.

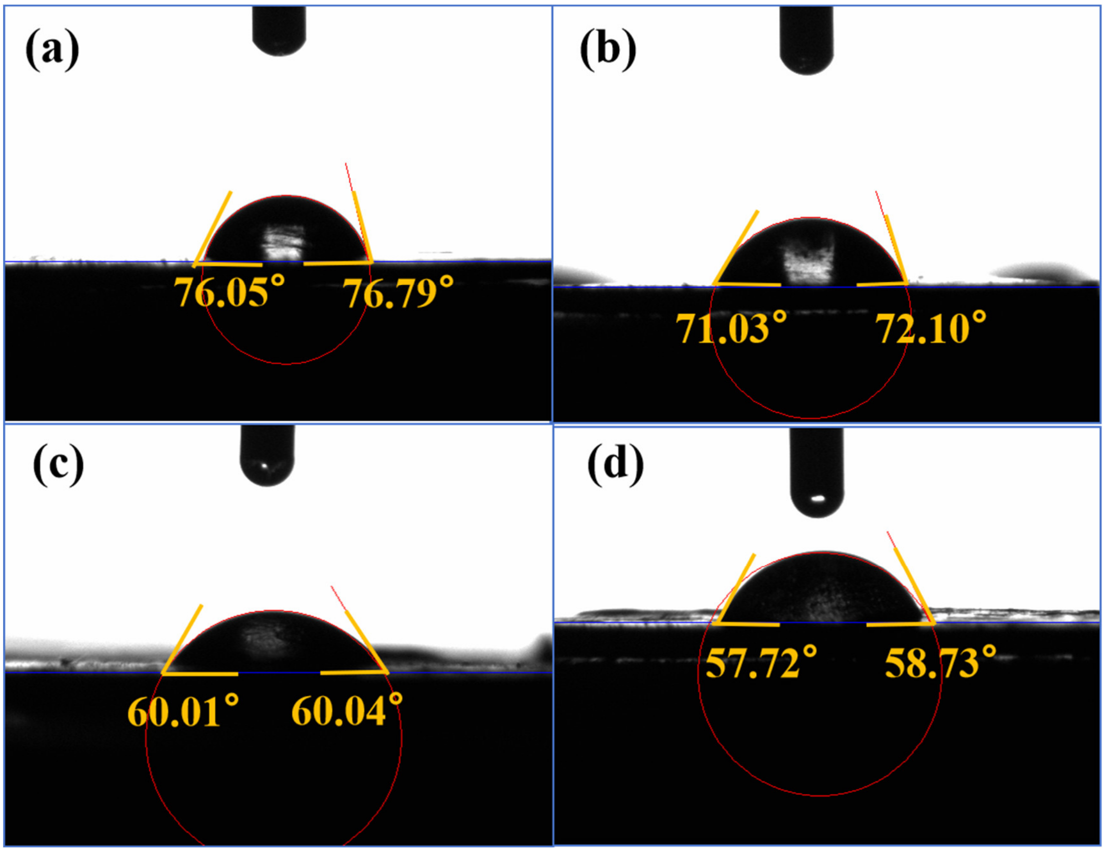

Figure S**3.** Contact angle test images of La-CNS$_x$: (a) La-CNS$_1$, (b) La-CNS$_2$, (c) La-CNS$_4$, (d) La-CNS$_5$..

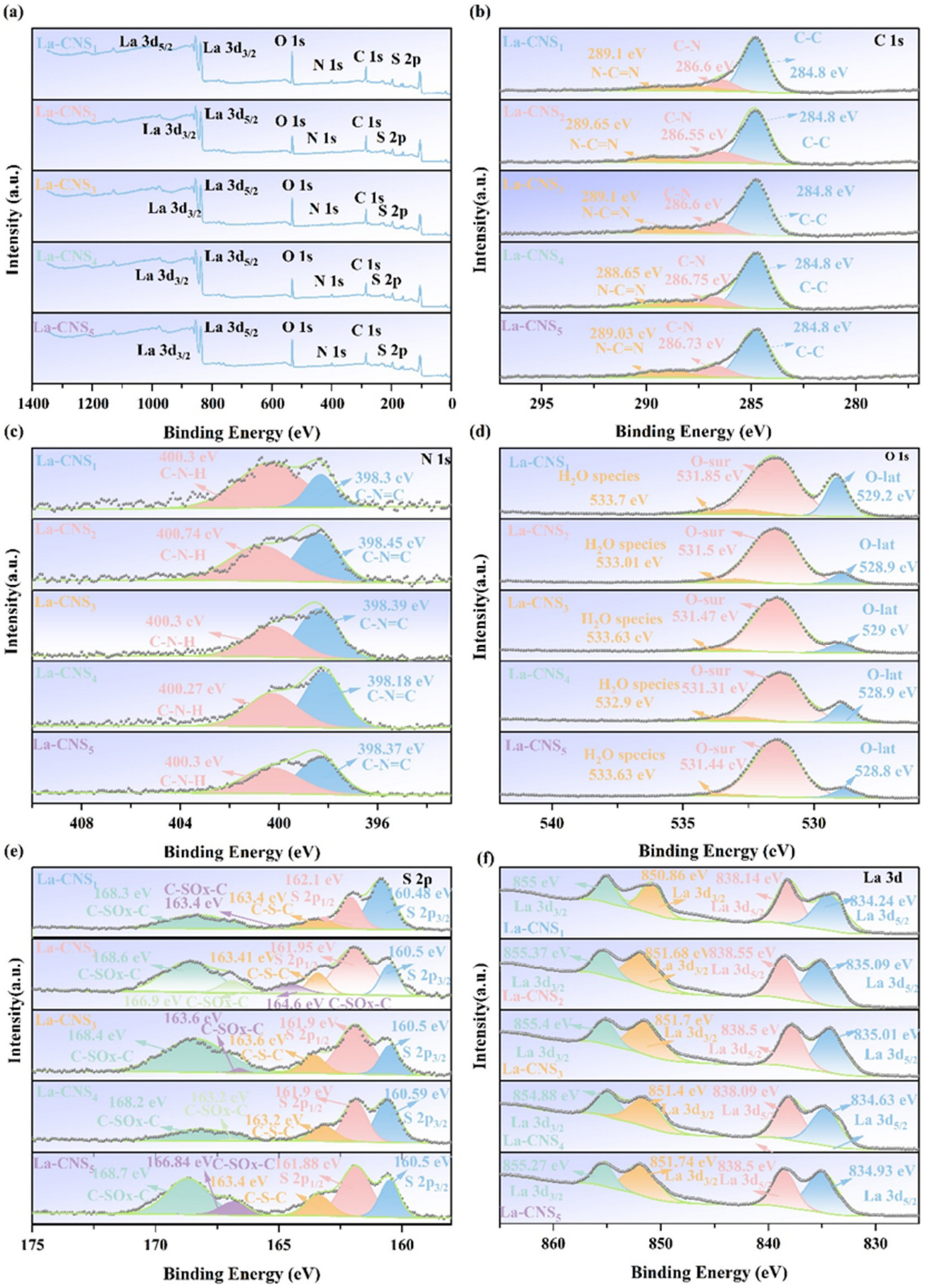

Figure S4. XPS spectra of La-CNSx: (a) O 1s spectra; (b) S 2p spectra; and (c) La 3d spectra.

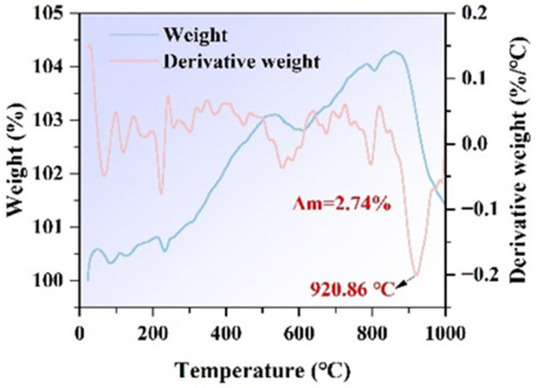

Figure S5. TG and DTG curves of La-CNS_3.

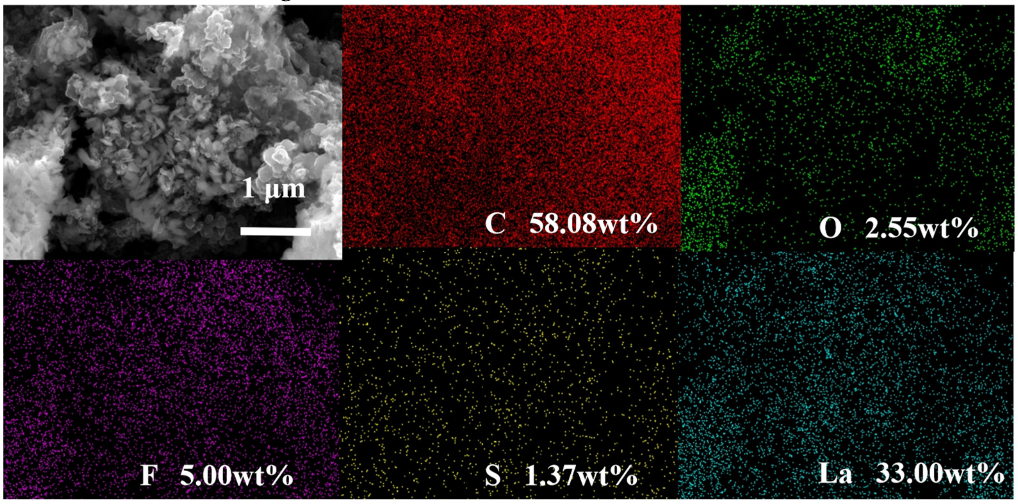

Figure S6. SEM images and EDS mapping of La-CNS_3 after 40 cycles.

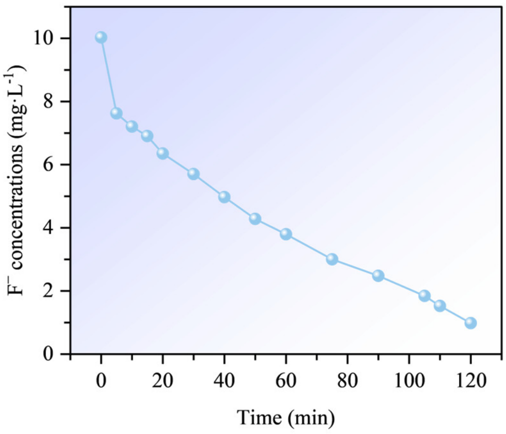

Figure S7. Variation of F^- concentration with time during static adsorption and under an applied voltage of 1.4 V at an initial F^- concentration of 10 mg·L^{-1}.

Table S1. Comparison of the fluoride adsorption capacity and regeneration efficiency of La-CNS_3 with recently reported CDI-derived carbon materials.

Electrode materials	Initial F^- concentration (mg·L^{-1})	q_e (mg·g^{-1})	Voltage (V)	Number of cycles	Regeneration efficiency	Ref.
CeO2@C	50	24.4	1.2	10	75	[1]
FeNx/C	100	158.33	1.2	3	87.5	[2]
Mg-Al-BC	/	19.6	1.4	20	97.8	[3]
MMCNF	10	20.64	1.2	5000	93.13	[4]
La-CNS_3	100	195	1.4	/	/	This work
	10	31.86	1.4	50	55	

Table S2 Parameters of the collected groundwater samples

Parameter	F^- (mg·L^{-1})	Cl^- (mg·L^{-1})	NO_3^- (mg·L^{-1})	SO_4^{2-} (mg·L^{-1})	pH
Concentration	0.44	18.98	2.68	46.86	7.45

[1] H. Kang, Z.Z. Lu, D. Zhang, H.S. Zhao, D.D. Yang, Z.N. Wang, Y.M. Li, Efficient fluoride removal in hybrid capacitive deionization enabled by Ce-Zn-MOF-derived CeO_2@C and birnessite electrodes, Sep. Purif. Technol., 353 (2025) 11.
[2] Q. Huang, S. Xie, L. Sheng, L. Huang, J. Yan, Z. Chen, M. Li, H. Zhang, High-performance fluoride removal by Fe/N co-doped microporous carbon: Mechanism of capacitive deionization with FeNx sites, Sep. Purif. Technol., 357 (2025) 130171.
[3] G. Wang, D. Chen, Z. Yang, S. Liao, R.S. Tamjidur, S. Hu, Q. Wu, W. Zhang, Effectiveness of capacitive deionization for the removal of soluble phosphorus and fluoride with a Mg/Al co-doped porous biochar electrode during the process of water-washing of phosphogypsum, Desalination, 602 (2025) 118640.
[4] X. Zhang, J. An, D. Zhang, In situ growth of Mg-MOF-derived dendritic carbon on lignin nanofibers for efficient capacitive defluorination, Desalination, 600 (2025) 118533.
